# Laser-cut stent-assisted coil embolization for basilar apex aneurysms: a retrospective single-center study

**DOI:** 10.1016/j.clinsp.2026.101011

**Published:** 2026-05-28

**Authors:** Chuanfu Chen, Lingfeng Lai, Jierong Zou, Qinfeng Chen, Hongji Zhu, Jing Huang, Zhiqun Jiang

**Affiliations:** a Department of Neurosurgery, the 1st Affiliated Hospital, Jiangxi Medical College, Nanchang University, Nanchang, Jiangxi, China; b Department of intervention center, Central People's Hospital of Ji'an, Ji'an, Jiangxi, China

**Keywords:** Intracranial aneurysm, Basilar apex aneurysms, Laser-cut stent, Coiling embolization, Retrospective study

## Abstract

**Objectives:**

To describe procedural and early outcomes following laser-cut Stent-Assisted Coil Embolization (SACE) for Basilar Apex Aneurysms (BAAs) at a single institution.

**Methods:**

The authors conducted a retrospective review of 43 consecutive patients with BAAs (both ruptured and unruptured) who underwent laser-cut SACE between September 2019 and April 2023. Outcomes included procedural success (defined as successful stent deployment and coil embolization without procedural abandonment or device malfunction), immediate and 6-month angiographic occlusion status (Raymond classification), periprocedural complications, and 3-month clinical status (modified Rankin Scale, mRS). Treatment strategies (single-stent vs. Y-configuration) and stent types (Enterprise vs. Atlas) were selected per operator discretion.

**Results:**

The procedural success rate was 100%. Immediate post-procedural angiography showed complete occlusion (Raymond I) in 81.4% (35/43) and neck remnant (Raymond II) in 18.6% (8/43). The periprocedural complication rate was 9.3% (4/43), including two thromboembolic events (4.7%), one fatal brain herniation, and one other non-specified complication. Among 42 surviving patients, 88.1% (37/42) had an mRS score ≤ 2 at 3-months. Six-month angiographic follow-up, available for 37 patients (86.0%), revealed complete occlusion in 97.3% (36/37) and neck remnant in 2.7% (1/37). Subgroup analyses comparing single-stent (*n* = 38) and Y-stent (*n* = 5) techniques, as well as Enterprise (*n* = 21) and Atlas (*n* = 17) stents in single-SACE, showed no statistically significant differences in occlusion rates or clinical outcomes, though these analyses were underpowered.

**Conclusion:**

In this single-center, retrospective experience of patients with saccular BAAs, laser-cut SACE for BAAs was associated with high procedural success and favorable early angiographic and clinical outcomes.

## Highlights

• Stent-assisted coiling is safe and effective for intracranial aneurysms.
• Safety and efficacy of laser-cut stents for basilar apex aneurysms were assessed.
• Y-stents may have lower recanalization rates versus single stents, without significance.
• No significant differences in outcomes were found between Atlas and Enterprise stents.

## Background

Basilar Apex Aneurysms (BAAs) account for 20%–40% of posterior circulation aneurysms.^1^ They typically present with rupture, mass effect, or thromboembolism and may also be discovered incidentally. While surgical treatments such as bypass and clipping were historically used for complex BAAs, these procedures are associated with high complication rates.^2^ Given the significant rupture risk of posterior circulation aneurysms, timely intervention is crucial.^3^^,^^4^ Despite advances in microsurgical techniques, clipping of BAAs remains challenging due to the complex local anatomy and the presence of critical surrounding vessels, leading to considerable morbidity and mortality. Excluding oculomotor nerve palsy, the postoperative complication rate for surgical clipping of BAAs is as high as 19.4%.^1^^,^^5^

Endovascular treatment has become the preferred approach for BAAs, offering lower complication rates.^6^ The main endovascular options are coil embolization and Stent-Assisted Coil Embolization (SACE). The introduction of Guglielmi detachable coils in the 1990s marked a major milestone in intracranial aneurysm treatment.^7^ Since the development of wide-neck BAA coiling in 2004, the Y-configuration double-stent-assisted technique has become a common procedure.^8^ Over the past decade, laser-cut stents have advanced significantly as key tools for embolization support. Standalone coiling has a high recanalization rate for basilar artery aneurysms, which is a major drawback.^9^ Stent assistance enhances flow remodeling, promotes intra-aneurysmal thrombosis, and encourages endothelialization at the neck, thereby reducing recanalization.^10^ Laser-cut stents (e.g., Enterprise, Atlas) are now considered standard for complex BAAs, providing superior radial support and flow diversion compared to braided designs.^11^ Their uniform nitinol mesh reduces wall shear stress, while their precise cell geometry adapts well to the basilar bifurcation anatomy ‒ advantages particularly important for wide-neck aneurysms.

Since the first retrospective analysis of intracranial stent placement in 2013, the number of SACE studies has increased,^12^ driven by growing stent implantation experience, technological improvements, and expanded indications for BAAs. However, existing studies on SACE for BAAs have several limitations: 1) Limited data on the procedural success and acute safety of modern laser-cut stents (e.g., Enterprise, Atlas); 2) Short follow-up durations (< 6-months) and small sample sizes, resulting in inconsistent reports on long-term occlusion durability; and 3) Insufficient comparison between single-stent and Y-stent techniques or between different devices.^13^^,^^14^ This study aims to address these gaps by evaluating laser-cut SACE in a consecutive series of BAA patients with 6-month angiographic follow-up. The study period (September 2019 to April 2023) was selected because September 2019 marked the institutional adoption of laser-cut stents (Enterprise, Atlas) as the standard device for BAAs, replacing prior non-laser-cut or braided designs; April 2023 was chosen as the cutoff to guarantee a minimum 6-month follow-up window for all patients, enabling consistent assessment of early angiographic outcomes. The authors confirm that no generative AI or AI-assisted technologies were used in this work, as clarified in the TITAN Guideline Checklist 2025.^15^

## Methods

### Study design and patient population

This single-center retrospective observational study was conducted in accordance with the Declaration of Helsinki. The study involved analysis of anonymized clinical and imaging data collected as part of routine clinical care, with no research-related interventions. Under institutional guidelines, retrospective studies using fully anonymized data may receive expedited ethics approval after data collection. Approval was granted retrospectively in August 2025 (approval number: IIT2025–644) prior to any data analysis or manuscript preparation. Due to the retrospective use of anonymized data and the absence of research-related interventions, the requirement for informed consent was waived. For patients presenting with acute subarachnoid hemorrhage, emergency endovascular treatment was performed after obtaining informed consent for the procedure from the patient's legal surrogate (next of kin), as the patients were incapacitated at presentation. This consent for treatment is distinct from the waiver of consent for retrospective data analysis described above. For patients who later recovered capacity, they were informed of the treatment received. No patient or surrogate objected to the use of anonymized data for research purposes. The study was performed at the 1st Affiliated Hospital, Jiangxi Medical College, Nanchang University, China. All patients were of East Asian (Han Chinese) ethnicity, reflecting the local demographic. This retrospective observational study is reported in accordance with the STROBE (Strengthening the Reporting of Observational Studies in Epidemiology) guidelines for cohort studies. The PROCESS criteria for case series reporting were also consulted, and a completed STROBE checklist is provided as Supplementary Material.^16^ The authors screened the institutional neurointerventional database to identify patients with BAAs treated between September 2019 and April 2023. From an initial 95 potential cases, 43 patients were included based on the following criteria: 1) Saccular BAA, 2) Treatment with laser-cut SACE using Enterprise or Atlas stents, and 3) Availability of baseline demographic, clinical, and procedural data. Exclusion criteria included: 1) Non-saccular BAAs (e.g., fusiform, dissecting), 2) Treatment with other modalities (e.g., clipping, flow diversion, braided stents, or simple coiling), and 3) Incomplete baseline or procedural data. Among the 52 excluded cases, reasons included alternative treatments, surgical clipping, incomplete data, and non-saccular morphology. The final sample of 43 patients represents the complete consecutive series of all eligible patients meeting the inclusion criteria during the study period; no eligible patient was excluded for any other reason. No a priori sample size calculation was performed, as this was an all-inclusive descriptive case series rather than a hypothesis-driven comparative trial. The sample size was therefore determined by the natural incidence of BAAs treated with laser-cut SACE at this institution over the 44-month study window.

### Endovascular procedures

All procedures were performed under general anesthesia. During the acute phase of Subarachnoid Hemorrhage (SAH), 23 patients underwent immediate intervention. The remaining 20 patients with unruptured aneurysms received elective treatment after completing preoperative antiplatelet therapy. For unruptured cases, dual antiplatelet therapy (clopidogrel 75 mg + aspirin 100 mg) was initiated 3–5 days before the procedure. For ruptured cases, intravenous tirofiban (8 μg/kg bolus) was administered perioperatively as an antiplatelet bridge, with infusion discontinued 2–4 h post-procedure to balance thrombotic and bleeding risks.^17^^,^^18^ Intraoperative anticoagulation was monitored via activated clotting time (target 250–300 s). Postoperative management included routine neurological assessments, platelet function testing (VerifyNow P2Y12 assay) in stable patients, and head CT within 24-hours to detect hemorrhagic complications. In cases of significant bleeding, platelet transfusion or desmopressin was available. To minimize rebleeding risk, coil embolization was prioritized before stent deployment in ruptured aneurysms.

All aneurysms were treated with bare platinum coils. Preoperative angiographic evaluation was performed using a Siemens AXION-Artis VB35D DSA system. A total of 48 laser-cut stents were implanted: 27 Enterprise (Codman) and 21 Atlas (Stryker) stents. Stent selection was individualized based on vascular anatomy and aneurysm morphology: Enterprise stents were preferred for tortuous vessels (> 45° angulation) or narrow lumens due to their superior trackability^19^; Atlas stents were used in straighter segments to optimize wall apposition and neck coverage. No standardized algorithm was applied; the choice was made intraoperatively based on real-time assessment.

Procedural complexity was quantified using: 1) Vascular tortuosity (maximum inter-segment angle > 45° on DSA), 2) Aneurysm neck width (mean 4.9 mm, range 1.5–10.6 mm; ≥ 4 mm defined as wide-neck), and 3) Bifurcation symmetry (angle difference between bilateral PCAs > 15° defined as asymmetry). Treatment planning integrated these parameters: the single-stent technique was used for neck width < 4 mm, asymmetric bifurcations, or severe tortuosity (> 60°); the Y-stent technique was reserved for neck width ≥4 mm, symmetric bifurcations, and centrally located necks at the basilar bifurcation. Five patients underwent Y-stent-assisted embolization: two with combined Enterprise/Atlas stents, two with dual Enterprise stents, and one with dual Atlas stents.

### Postoperative management

All patients received at least 1.5 months of dual antiplatelet therapy followed by aspirin for 6-months, with adjustments based on follow-up evaluations. Preoperative platelet function was assessed using VerifyNow P2Y12 assay (PRU < 208 defined adequate inhibition)^20^ and Thromboelastography (TEG) (maximum amplitude < 50 mm indicated sufficient inhibition).^21^ CYP2C19 genotyping guided antiplatelet selection in poor metabolizers.

### Data collection

Data were extracted from medical records, imaging archives, and angiographic studies. Collected variables included demographics, comorbidities (hypertension, diabetes, dyslipidemia), smoking status, admission details, SAH occurrence, Hunt-Hess grade, modified Rankin Scale (mRS) score, hospital length of stay, and mortality. Aneurysm characteristics (diameter, neck width, multiplicity) and procedural details (technique, complications, angiographic outcomes) were also recorded.

### Outcome definition and follow-up

Angiographic outcomes were assessed immediately post-procedure and at 6-month follow-up using the Raymond classification: complete occlusion (grade I), residual neck (grade II), or residual aneurysm (grade III).^22^ Clinical outcomes were evaluated via mRS at 3-months postoperatively, dichotomized as good outcome (mRS ≤ 2) or dependent survival (mRS > 2). Complications were identified clinically (new/worsening NIHSS score, hemorrhage), radiologically, and classified per Society of Interventional Radiology criteria. Follow-up at 3- and 6-months was conducted via structured telephone interviews by trained nurses using a standardized mRS script. To ensure reliability, ambiguous responses underwent secondary assessment within 48-hours, unclear cases were re-interviewed within one week, and unreachable patients received three contact attempts. Clinical records were reviewed for 5% of cases to validate data completeness.

### Statistical analysis

Continuous variables are presented as means with Standard Deviations (SD) and ranges. Categorical variables are presented as frequencies with percentages. Group comparisons were performed using independent-sample *t*-tests for continuous variables and chi-square or Fisher’s exact tests for categorical variables. All tests were two-tailed, with statistical significance set at *p* < 0.05. Analyses were conducted using SPSS version 26.0 (IBM Corp., Armonk, NY, USA).

## Results

### Patient characteristics

The study cohort comprised 43 patients, of whom 69.8% (*n* = 30) were female. The mean age was 59-years (SD = 12.6 years; range: 33–87 years). The mean aneurysm neck width was 4.9 mm (SD = 2.0 mm; range: 1.5–10.6 mm). Ruptured aneurysms accounted for 53.5% (*n* = 23) of cases. Demographic and aneurysmal characteristics are summarized in Table 1. Thromboembolic events occurred in 4.7% (*n* = 2) of patients, while a poor clinical outcome (mRS score > 2) was observed in 11.9% (*n* = 5/42). The observed rate of thromboembolic events was lower than anticipated; therefore, the study was underpowered to identify predictors of thromboembolism or unfavorable outcomes.

**Table 1:** Baseline characteristics of included patients.

Variables	n (%)
Age (years)	
< 50	8 (18.6%)
≥ 50	35 (81.4%)
Female	30 (69.8%)
Smoking	13 (30.2%)
Underlying diseases	
Hypertension	22 (51.2%)
Diabetes mellitus	14 (32.6%)
Dyslipidemia	10 (23.3%)
Initial presentation	
Subarachnoid hemorrhage	23 (53.5%)
Mass effect	1 (2.3%)
Incidental finding	19 (44.2%)
Hunt and Hess grade	6 (14.0%)
Good (1, 2 or 3)	17 (39.5%)
Poor (4 or 5)	6 (14.0%)
Preoperative comorbidity	
Hydrocephalus	2 (4.7%)
Aneurysm size	
Small (< 10 mm)	36 (83.7%)
Large (10–24 mm)	7 (16.3%)
Unruptured	20 (46.5%)
Wide neck	32 (74.4%)
Aneurysm configuration	
Saccular	38 (88.4%)
Daughter sac	5 (11.6%)
Specific condition	
MMD	9 (20.9%)
Multiple aneurysms	17 (39.5%)
Immediate post-procedural angiographic outcome	
Complete occlusion	35 (81.4%)
Neck remnant	8 (18.6%)
Incomplete occlusion	0 (0.0%)
Treatment type	
Single stent	38 (88.4%)
Y-stent	5 (11.6%)
Stent type	
Enterprise	27 (62.8%)
Atlas	21 (48.8%)

Single stent, Single stent-assisted coiling; MMD, Moyamoya Disease; Y-stent, Y-configuration double-stent-assisted coiling.

### Clinical and angiography follow-up

**Fig. 1:**
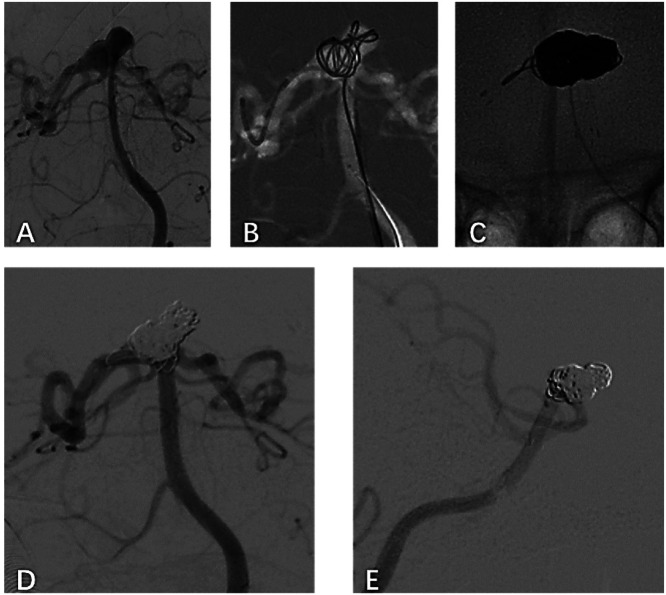
The frontal view (A) of the left vertebral artery injection Digital Subtraction Angiography (DSA) shows a 10-mm wide-necked basilar apex aneurysm. The stent catheter was advanced under roadmap guidance to the P2 segment of the posterior cerebral artery and filled with the first 3D spring coil (B). The Enterprise stent was deployed from the basilar trunk into the left posterior cerebral artery (C). The frontal view of the left vertebral artery injection DSA shows almost complete occlusion of the aneurysm after spring coil embolization (D) and progression to complete occlusion at the 6-month follow-up angiography (E).

**Fig. 2:**
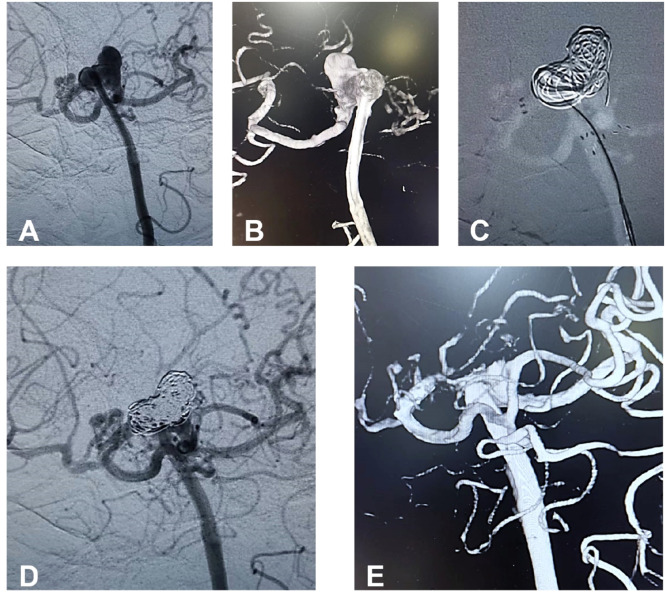
The frontal view (A) and 3D reconstruction (B) of the left vertebral artery Digital Subtraction Angiography (DSA) show a 9-mm wide basilar apex aneurysm with two Posterior Cerebral Arteries (PCA). Dual Atlas stents were deployed in a Y-shaped configuration within the basilar trunk and bilateral PCA (C). The frontal view of the DSA injected into the left vertebral artery showed no filling of the aneurysm following coil embolization (D). The 6-month follow-up angiography (E) revealed complete occlusion of the BTA.

Follow-up data were available for all 42 patients. Clinical outcomes assessed at 3-months postoperatively showed that 37 patients (88.1%) achieved independent living (mRS ≤ 2), while five patients (11.9%) required assistance (mRS > 2). Immediate postprocedural angiography demonstrated complete aneurysm occlusion (Raymond grade I) in 81.4% (*n* = 35) of patients and near-complete occlusion (Raymond grade II) in 18.6% (*n* = 8). Among the 37 patients (86.0%) who underwent 6-month follow-up digital subtraction angiography, complete occlusion (Raymond grade I) was achieved in 97.3% (*n* = 36); one patient (2.7%) showed residual neck filling (Raymond grade II) and was recommended for further treatment (Fig. 1, Fig. 2).

### Exploratory analysis

Patients were stratified by endovascular technique into single-stent-assisted coiling and Y-configuration double-stent-assisted coiling groups. Angiographic and clinical outcomes are presented in Table 2. No significant differences were observed between the two groups in immediate Raymond grade (*p* = 0.212), perioperative complications (*p* = 0.835), 3-month mRS (*p* = 0.688), or 6-month Raymond grade (*p* = 0.703). Although no short-term differences were statistically significant, the Y-stent technique was selectively applied to anatomically complex cases to optimize long-term flow remodeling. Hemodynamic studies suggest that Y-stent constructs may enhance late occlusion durability, though longer follow-up is required to confirm this in the present cohort.

**Table 2:** Angiographic and clinical outcomes of basilar tip aneurysms treated with different endovascular techniques.

	Single stent, n (%)	Y-stent, n (%)	All Stent Techniques, n (%)	p-value
Initial Raymond 1	32 (84.2%)	3 (60.0%)	35 (81.4%)	0.212
Initial Raymond 2	6 (15.8%)	2 (40.0%)	8 (18.6%)	
Thromboembolic complications	2 (5.3%)	0 (0%)	2 (4.7%)	0.835
Last follow-up Raymond 1	31 (96.9%)	5 (100.0%)	36 (97.3%)	0.703
Last follow-up Raymond 2	1 (3.1%)	0 (0%)	1 (2.7%)	
mRS of follow-up (0‒2)	32 (86.5%)	5 (100%)	36 (88.1%)	0.688
mRS of follow-up (3‒6)	5 (13.5%)	0 (0%)	5 (11.9%)	

Single stent, Single stent-assisted coiling; Y-stent, Y-configuration double-stent-assisted coiling.

To compare the performance of different laser-cut stents in single-stent procedures, patients were further divided into Enterprise (*n* = 21) and Atlas (*n* = 17) stent groups. Baseline characteristics and outcomes are shown in Table 3. The Enterprise group was significantly older (mean age 67.52 vs. 57.82 years, *p* < 0.001), had a higher prevalence of hypertension (81.0%vs. 23.5%, *p* < 0.001), and a greater proportion of ruptured aneurysms (76.2%vs. 35.3%, *p* = 0.011). Aneurysm neck width did not differ significantly between groups (5.26 vs. 4.19 mm, *p* = 0.067). No significant between-group differences were found in thromboembolic events (*p* = 0.878), immediate Raymond grade I occlusion (*p* = 0.540), mortality (*p* = 0.362), recanalization (*p* = 0.360), 6-month Raymond grade I occlusion (*p* = 0.279), or favorable 3-month mRS (0–2) (*p* = 0.211). Due to the limited sample size, multivariate adjustment for baseline imbalances was not performed. However, the absence of significant outcome differences despite these disparities suggests potential robustness of the findings.

**Table 3:** Comparison of different laser-cut stents in the treatment of basilar tip aneurysms.

	Laser-Cut Stent		p-value
	Enterprise	Atlas	
Number of patients	21	17	N/A
Age, y	67.52±12.60	51.82±9.05	<0.01*
Female sex, n/N (%)	16/21 (76.2%)	10/17 (58.8%)	0.252
Hypertension, n/N (%)	17/21 (81.0%)	4/17 (23.5%)	<0.01*
Aneurysm neck size, mm	5.2610±1.83	4.1859±2.29	0.067
Operation duration, min	164.62±47.34	152.76±54.29	0.521
Ruptured aneurysms, n/N (%)	16/21 (76.2%)	6/17 (35.3%)	0.011
Thromboembolic events, n/N (%)	1/21 (4.8%)	1/17 (5.9%)	0.878
Initial Raymond 1, n/N (%)	17/21 (81.0%)	15/17 (88.2%)	0.540
Mortality, n/N (%)	1/21 (4.8%)	0/17 (0%)	0.362
Recanalization, n/N (%)	0/20^a^ (0%)	1/17 (5.9%)	0.360
Last follow-up Raymond I, n/N (%)	17/17^b^ (100.0%)	14/15^c^ (93.3%)	0.279
mRS of follow-up (0‒2), n/N (%)	16/20^a^ (80.0%)	16/17 (94.1%)	0.211
Recanalization, n/N (%)	0/20^a^ (0%)	1/17 (5.9%)	0.360

mRS, modified Rankin Scale; DSA, Digital Subtraction Angiography.

⁎ Statistically significant. mRS, modified Rankin Scale.

a 1-patient died; ^b^ 17-cases were reviewed for DSA; ^c^ 15-cases were reviewed for DSA.

## Discussion

The management of BAAs poses significant challenges, primarily due to the small caliber of parent vessels and the typically wide aneurysm neck. Endovascular intervention has emerged as the preferred treatment, offering a minimally invasive approach to achieve aneurysm occlusion through stent-, coil-, or flow-diverter-based techniques.^23^

The selection of Enterprise and Atlas laser-cut stents in this study was guided by their favorable biomechanical profile: high conformability facilitates adaptation to the tortuous cerebrovascular anatomy, excellent flexibility enables navigation through curved segments, and reliable radial stability provides durable support for coil embolization. These characteristics collectively contribute to a lower risk of peri‑procedural thromboembolism. In contrast, other laser-cut stents may offer less optimal balance of these properties, potentially leading to suboptimal apposition, higher recanalization rates, or technical failure in complex anatomies.

The single-center retrospective experience demonstrates that laser-cut SACE is procedurally feasible, with a 100% success rate for stent deployment and coil embolization without procedural abandonment or device malfunction, and a 6-month complete occlusion rate of 97.3% among patients with follow-up imaging. These encouraging results should be interpreted in light of the study’s limitations, including its non-comparative design, operator-dependent treatment selection, small sample size (particularly for the Y-stent subgroup), and limited follow-up duration. The observed outcomes may be attributed to several factors: 1) Increasing operator familiarity with laser-cut stent technology and refined procedural skills^24^; 2) Iterative improvements in stent design, including more precise deployment mechanisms and enhanced biocompatibilit;y^8^ and 3) Advances in neuroimaging allowing more accurate anatomical assessment and procedural planning.

In the evolving landscape of posterior circulation aneurysm treatment, laser-cut SACE offers distinct advantages over flow diverters in selected scenarios: 1) For ruptured aneurysms (57.9% in the studied cohort), SACE provides immediate occlusion without the need for prolonged antiplatelet therapy, which is often deferred in flow-diverter cases^25^; 2) For saccular BAAs with neck width < 6 mm, SACE achieved a 97.3% 6-month occlusion rate in this series, demonstrating efficacy comparable to that reported for flow diversion^26^; and 3) SACE may be associated with a lower risk of delayed ischemic events, rendering it preferable in elderly patients or those with comorbid cerebrovascular disease.^27^

Stent-assisted coiling mitigates several established risk factors for recanalization, such as wide neck and large aneurysm volume, by providing scaffold support to prevent coil compaction and promoting intra-aneurysmal thrombosis and endothelialization.^9^^,^^10^ Although stent use entails inherent risks of acute thrombosis, peri‑procedural antiplatelet management, as implemented in this cohort, effectively minimizes such complications.^28^ In the present series, thromboembolic events occurred in 4.6% of patients, consistent with prior reports,^29^ and none resulted in permanent neurological deficits.

Both single-stent and Y-stent configurations yielded favorable angiographic and clinical outcomes without significant short-term differences.^30^ The numerically lower recanalization rate in the Y-stent subgroup (0%vs. 3.1%) may reflect its dual-stent scaffolding effect and enhanced flow diversion, which promote more stable occlusion.^31^^,^^32^ Hemodynamic studies further suggest that Y-stent constructs induce favorable vascular remodeling, potentially improving long-term durability.^31^^,^^32^ Anatomically, Y-stenting is particularly warranted for 1) Bifurcation-centered aneurysms requiring protection of both posterior cerebral arteries, 2) Wide-neck lesions (≥ 4 mm) where enhanced flow diversion is needed, and 3) Symmetric bifurcations requiring balanced stent coverage.

Subgroup analysis of single-stent cases (Enterprise vs. Atlas) revealed significant baseline differences: the Enterprise group was older, had higher hypertension prevalence, and more frequently presented with rupture. Despite these higher-risk features, occlusion rates and complication profiles did not differ between stent types, suggesting comparable performance across heterogeneous patient populations. These findings underscore the safety and feasibility of laser-cut SACE even in clinically complex scenarios.

This study has several important limitations: 1) Its retrospective, non-comparative design precludes causal inference and introduces potential selection bias; 2) The small, heterogeneous sample ‒ especially the limited Y-stent subgroup (*n* = 5) ‒ constrains statistical power and generalizability; 3) Treatment allocation was based on operator preference and anatomical factors, leading to baseline imbalances that were not adjusted for; 4) The 6-month angiographic follow-up is insufficient to assess long-term durability, and follow-up was incomplete; 5) Outcome assessment lacked independent core-lab adjudication, introducing potential ascertainment bias; and 6) The study was underpowered for subgroup comparisons, rendering negative findings inconclusive.

In this retrospective series, laser-cut SACE for BAAs was technically feasible and associated with high rates of early complete occlusion and favorable short-term clinical outcomes. However, the non-comparative design, small sample size, treatment selection bias, and limited follow-up preclude definitive conclusions regarding its efficacy, safety, or comparative performance relative to other endovascular strategies. These results underscore the need for prospective, multicenter studies with longer follow-up to establish evidence-based treatment algorithms for complex BAAs.

## Statement

Ethical approval was obtained from the central ethics committee of the 1st Affiliated Hospital, Jiangxi Medical College, Nanchang University (n°IIT2025–644). All data were collected and analyzed in a fully anonymized manner in compliance with institutional and national data protection regulations. The individual patient consent was waived due to the retrospective design and the use of fully anonymized data.

## Data availability

The datasets generated and/or analyzed during the current study are available from the corresponding author upon reasonable request.

## Authors' contributions

Study conception: CC, LL, and JZ; Data collection: CC, LL, ZJ, CQ, ZH, and HJ; Manuscript preparation: CC, and LL; Critical review and revision: all authors; Final approval of the article: all authors; Accountability for all aspects of the work: all authors.

## Funding

This study funded by the Investigator Initiated Trial project of the First Affiliated Hospital of 10.13039/501100004637Nanchang University (Number: 2021039).

## Declaration of competing interest

The authors declare no conflicts of interest.

## Appendix Supplementary materials

Image, application 1

Image, application 2
